# The potential for improving cardio-renal outcomes in chronic kidney disease with the aldosterone synthase inhibitor vicadrostat (BI 690517): a rationale for the EASi-KIDNEY trial

**DOI:** 10.1093/ndt/gfae263

**Published:** 2024-11-12

**Authors:** Parminder K Judge, Katherine R Tuttle, Natalie Staplin, Sibylle J Hauske, Doreen Zhu, Rebecca Sardell, Lisa Cronin, Jennifer B Green, Nikita Agrawal, Ryoki Arimoto, Kaitlin J Mayne, Emily Sammons, Martina Brueckmann, Shimoli V Shah, Peter Rossing, Masaomi Nangaku, Martin J Landray, Christoph Wanner, Colin Baigent, Richard Haynes, William G Herrington

**Affiliations:** Renal Studies Group, Clinical Trial Service Unit & Epidemiological Studies Unit, Nuffield Department of Population Health, University of Oxford, Oxford, UK; Providence Inland Northwest Health, Spokane, WA and University of Washington, Seattle, WA, USA; Renal Studies Group, Clinical Trial Service Unit & Epidemiological Studies Unit, Nuffield Department of Population Health, University of Oxford, Oxford, UK; Boehringer Ingelheim International GmbH, Ingelheim, Germany; Vth Department of Medicine, University Medical Center Mannheim, Heidelberg University, Mannheim, Germany; Renal Studies Group, Clinical Trial Service Unit & Epidemiological Studies Unit, Nuffield Department of Population Health, University of Oxford, Oxford, UK; Renal Studies Group, Clinical Trial Service Unit & Epidemiological Studies Unit, Nuffield Department of Population Health, University of Oxford, Oxford, UK; Boehringer Ingelheim International GmbH, Ingelheim, Germany; Duke Clinical Research Institute, Durham, NC, USA; Renal Studies Group, Clinical Trial Service Unit & Epidemiological Studies Unit, Nuffield Department of Population Health, University of Oxford, Oxford, UK; Renal Studies Group, Clinical Trial Service Unit & Epidemiological Studies Unit, Nuffield Department of Population Health, University of Oxford, Oxford, UK; Renal Studies Group, Clinical Trial Service Unit & Epidemiological Studies Unit, Nuffield Department of Population Health, University of Oxford, Oxford, UK; School of Cardiovascular and Metabolic Health, College of Medical and Veterinary Life Sciences, University of Glasgow, Glasgow, UK; Renal Studies Group, Clinical Trial Service Unit & Epidemiological Studies Unit, Nuffield Department of Population Health, University of Oxford, Oxford, UK; Boehringer Ingelheim International GmbH, Ingelheim, Germany; Ist Department of Medicine, University Medical Center Mannheim, Heidelberg University, Mannheim, Germany; Boehringer Ingelheim International GmbH, Ingelheim, Germany; Steno Diabetes Center Copenhagen, Copenhagen, Denmark; Department of Clinical Medicine, University of Copenhagen, Copenhagen, Denmark; Division of Nephrology and Endocrinology, University of Tokyo Hospital, Tokyo, Japan; Renal Studies Group, Clinical Trial Service Unit & Epidemiological Studies Unit, Nuffield Department of Population Health, University of Oxford, Oxford, UK; Renal Studies Group, Clinical Trial Service Unit & Epidemiological Studies Unit, Nuffield Department of Population Health, University of Oxford, Oxford, UK; University Clinic of Würzburg, Würzburg, Germany; Renal Studies Group, Clinical Trial Service Unit & Epidemiological Studies Unit, Nuffield Department of Population Health, University of Oxford, Oxford, UK; Renal Studies Group, Clinical Trial Service Unit & Epidemiological Studies Unit, Nuffield Department of Population Health, University of Oxford, Oxford, UK; Renal Studies Group, Clinical Trial Service Unit & Epidemiological Studies Unit, Nuffield Department of Population Health, University of Oxford, Oxford, UK

**Keywords:** aldosterone, cardiovascular, CKD, clinical trial, heart failure

## Abstract

Patients with chronic kidney disease (CKD) are at risk of progressive loss of kidney function, heart failure, and cardiovascular death despite current proven therapies, including renin-angiotensin system inhibitors (RASi), sodium glucose co-transporter-2 inhibitors (SGLT2i), and statin-based regimens. RASi and SGLT2i reduce risk of CKD progression irrespective of primary cause of kidney disease, suggesting they target final common pathways. Targeting aldosterone overactivity with a nonsteroidal mineralocorticoid receptor antagonist (MRA) also reduces cardiorenal risk in patients with albuminuric diabetic kidney disease already treated with RASi. Together, these observations provide the rationale for trials to assess effects of inhibiting the aldosterone pathway in a broader range of patients with CKD, including those with non-diabetic causes of CKD or low albuminuria. Aldosterone synthase inhibitors (ASi) have emerged as an alternative to MRAs for aldosterone pathway inhibition. Phase II data from 586 patients with albuminuric CKD have shown that 10 mg of an ASi, vicadrostat (BI 690517), reduced urine albumin-to-creatinine ratio by ∼40% compared with placebo, with or without concurrent empagliflozin treatment. MRA and ASi increase risk of hyperkalaemia. Combining their use with an SGLT2i may mitigate some of this risk, improving tolerability, and allowing a wider range of patients to be treated (including those with higher levels of blood potassium than in previous trials). The EASi-KIDNEY (NCT06531824) double-blind placebo-controlled trial will test this approach by assessing the safety and cardiorenal efficacy of vicadrostat in combination with empagliflozin in ∼11 000 patients with CKD. It will be sufficiently large to assess effects in patients with and without diabetes separately.

## Graphical Abstract

**Graphical Abstract:**
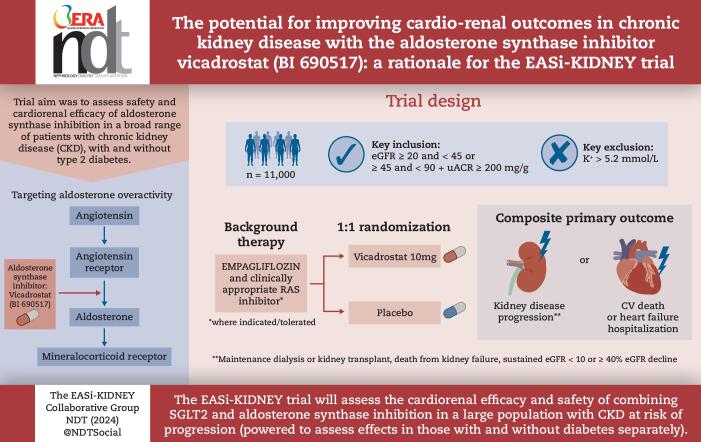

KEY LEARNING POINTS **What was known**:

- Targeting residual aldosterone overactivity despite maximally tolerated renin-angiotensin system inhibitor with a nonsteroidal mineralocorticoid receptor antagonist reduces risks of chronic kidney disease (CKD) progression and heart failure hospitalization in patients with CKD, albuminuria, and type 2 diabetes.
- Large-scale randomized trials are now needed to assess the effects of inhibiting the aldosterone pathway in the less studied types of patients with CKD, including those with non-diabetic causes, those with low albuminuria, and those with the somewhat higher levels of potassium excluded from previous trials.
- The aldosterone synthase inhibitor vicadrostat (BI 690517) 10 mg once daily reduces albuminuria by ∼40% and blood aldosterone levels by ∼60%, on top of the effects of SGLT2 inhibition.

**This study adds**:

- There is a rationale to study the effect of aldosterone synthase inhibitors in a broad range of patients with CKD at risk of progression.

**Potential impact**:

- The EASi-KIDNEY trial will be a definitive test of the efficacy and safety of the aldosterone synthase inhibitor vicadrostat in combination with empagliflozin in CKD, and is powered to assess effects in patients with and without diabetes separately.

## SUBSTANTIAL RESIDUAL RISK OF CKD PROGRESSION REMAINS DESPITE USE OF EFFECTIVE MEDICAL THERAPIES

In adults, the overall prevalence of CKD is ∼10%. There is regional variation, but the burden is spread across low, middle, and high income countries [1, 2]. CKD prevalence is likely to rise as the global average population age increases and diabetes becomes more prevalent [3, 4]. The avoidance of kidney failure is highly desirable due to its adverse effects on morbidity, quality-of-life, and the substantial costs of dialysis and transplantation [5]. Albuminuria represents a key predictor for more rapid decline in kidney function and cardiovascular disease [6]. Albuminuria may represent underlying kidney pathology and/or the presence of intraglomerular hypertension. Intraglomerular hypertension can result from obesity, diabetes, systemic hypertension, or a maladaptive glomerular haemodynamic response to reduced nephron numbers in CKD [7, 8]. The latter cause of intraglomerular hypertension is considered to be a final common pathway for progression of many forms of CKD. The existence of final common pathways is supported by key observations. First, for a given level of albuminuria, the risk of kidney failure appears to be relatively independent of the primary cause of CKD [9]. Second, renin-angiotensin system inhibitors (RASi) and sodium glucose co-transporter-2 inhibitors (SGLT2i) have been shown to reduce risk of CKD progression irrespective of primary kidney diagnosis [10–16].

**Figure 1:**
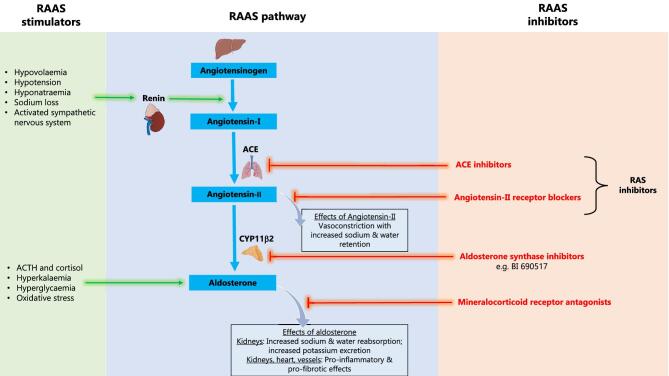
The renin-angiotensin-aldosterone system (RAAS) pathway and mechanisms of inhibition. Angiotensin-II causes sodium and water retention and increased blood pressure through several mechanisms including arteriolar vasoconstriction, increased sympathetic drive, hypothalamic thirst centre stimulation, posterior pituitary anti-diuretic hormone release, increased renal tubular sodium reabsorption, in addition to stimulating adrenal aldosterone synthesis. The activity of the renin-angiotensin system (RAS) is blocked by both ACE inhibitors and angiotensin-II receptor blockers. Classical genomic effects of aldosterone are mediated by aldosterone binding to cytoplasmic mineralocorticoid receptor (MR). Other modulators of the MR include sodium, caveolin-1, and Rac-1. Rapid non-genomic effects of aldosterone are modulated by intracellular second messenger molecules such as protein kinase C, epidermal growth factor receptor, and members of the mitogen-activated protein kinase family. The mechanism of interactions between genomic and non-genomic effects of aldosterone are unclear. Addition of MR antagonism has been shown to inhibit the effects of aldosterone, reducing albuminuria, risk of kidney disease progression, and heart failure hospitalization, but increases blood aldosterone levels. Aldosterone synthase inhibitors are an alternative method to target the effects of aldosterone excess.

The initial acute ‘dip’ in estimated glomerular filtration rate (eGFR) on initiating RASi or SGLT2i—which is reversible—is often attributed to reduction of intraglomerular hypertension [17]. In randomized trials, the acute dip with a RASi or SGLT2i is followed by a decrease in annual rate of long-term (or chronic) eGFR decline [7, 17, 18]. RASi are considered to achieve this effect by counteracting increased glomerular efferent arteriolar tone (mediated by angiotensin-II from RAS overactivity) [19]. Conversely, SGLT2i are considered to correct maladaptive reduced glomerular afferent arteriolar tone through restoration of tubuloglomerular feedback [16, 20, 21]. Combined use of RASi and SGLT2i is now part of a standard-of-care for CKD (Fig. 1) [22, 23]. Despite these interventions, eGFR decline is not fully arrested, and residual risk of kidney failure remains for many patients with CKD [16, 24].

## CKD IS ASSOCIATED WITH SUBSTANTIAL RISK OF CARDIOVASCULAR DISEASES, AND PARTICULARLY FROM HEART FAILURE

Cardiovascular risk increases progressively as kidney function declines [25, 26], and cardiovascular diseases are a leading cause of morbidity and mortality in patients with CKD [27–30]. In addition to increasing risk of atherosclerotic cardiovascular disease, as eGFR declines, prevalence of non-atherosclerotic cardiovascular disease characterized by arteriosclerosis and structural heart disease rises sharply [31, 32]. Studies have identified that about one half of patients with CKD stages G4–5 have abnormal cardiac structure on echocardiography [33, 34], increasing to over 80% by the time dialysis is initiated [34]. Clinically this manifests as both heart failure (HF) and a high incidence of sudden cardiac death [35]. Current management strategies include lowering low-density lipoprotein cholesterol (LDL-C) with a statin-based regimen [36], intensive blood pressure (BP) control [37], and widespread use of SGLT2i [16, 38]. The reductions in relative risk for HF hospitalization with use of SGLT2i are particularly large in patients with pre-existing HF irrespective of ejection fraction [16, 39]. In patients with type 2 diabetes and CKD with albuminuria, use of the glucagon-like peptide-1 (GLP-1) agonist semaglutide [40] and the non-steroidal mineralocorticoid receptor antagonist (MRA) finerenone [41, 42] also reduces cardiovascular risk. Nevertheless, there is a need for further trials to test interventions that could further reduce the cardiovascular risks associated with CKD.

## RECENT RENEWED INTEREST IN THE ALDOSTERONE SIGNALLING PATHWAY IN CKD

A feature of glomerular hyperfiltration and CKD is activation of the RAS [43, 44]. It has been long-established from randomized trials that RASi (i.e. an angiotensin-converting enzyme inhibitor [ACEi] or angiotensin-II receptor blocker [ARB]) reduce risk of kidney failure in patients with albuminuric CKD with and without diabetes, with particularly clear evidence in overt diabetic nephropathy [10–12]. ARBs also slow progression from microalbuminuria to overt nephropathy, supporting early treatment [11, 12]. The strategy of combining ACEi and ARB therapy reduces albuminuria more than either agent alone, but large randomized trials found that this did not translate into either additional renoprotective or cardiovascular benefits compared with single-agent RASi. Such ‘dual-blockade’ increased the risk of hyperkalaemia and acute kidney injury (AKI) [45, 46]. However, there is renewed interest in targeting overactivation of the renin-angiotensin-*aldosterone* system (RAAS) in CKD due to demonstrable kidney and cardiovascular benefits in large trials of the nonsteroidal MRA finerenone, which was tested on patients with CKD and type 2 diabetes (with albuminuria) who were treated with maximally tolerated doses of a RASi [41, 47, 48], and in HF with mildly reduced or preserved ejection fraction [49].

Aldosterone is a mineralocorticoid hormone, mainly synthesized in the zona glomerulosa of the adrenal cortex [50]. Aldosterone production is stimulated by RAS activation in response to hypotension, hyponatraemia, or hypovolaemia [51]. Production is also stimulated directly by angiotensin-II, hyperkalaemia, and adrenocorticotrophic hormone (ACTH). Aldosterone acts downstream of the RAS, forming the last effector component of this RAAS cascade (we use the term RAAS to refer to RAS plus the aldosterone part of the pathway). In addition to causing sodium and water retention, potassium excretion and increased BP, aldosterone is considered to be a key mediator of pro-inflammatory and profibrotic pathways, contributing to pathophysiology of both non-atherosclerotic cardiovascular disease (cardiac fibrosis, ventricular hypertrophy, and heart failure) and CKD (including glomerular hypertrophy, glomerulosclerosis, tubulointerstitial fibrosis, and vascular remodelling) (Fig. 1) [50, 52, 53].

Aldosterone exerts its classical genomic effects by activating the mineralocorticoid receptor (MR) [51], which is widely distributed in the kidneys, heart, vasculature, and fibroblasts. In the kidney, aldosterone acts via the MR in aldosterone-sensitive epithelial cells in the distal nephron, which control sodium and potassium balance [50]. The MR can also be activated by glucocorticoids, oxidative stress, and high salt intake [50, 54, 55]. Paradoxically, MR antagonism could trigger increased aldosterone levels [56]. Non-genomic effects of aldosterone have also been reported leading to increased levels of protein kinase C, intracellular calcium, epidermal growth factor receptor, and cyclic adenosine monophosphate, and increased generation of reactive oxygen species, which may mediate some of aldosterone's deleterious effects (Fig. 1) [57]. It remains unclear if these non-genomic effects of aldosterone are exerted via the MR and therefore it is uncertain as to whether inhibiting the MR impacts both genomic and non-genomic effects of aldosterone [57].

The particular effectiveness of MRAs in treating CKD in people with type 2 diabetes (with albuminuria), and HF [49, 58, 59] supports specifically targeting the aldosterone component of the RAAS cascade [41]. The Finerenone in Reducing Kidney Failure and Disease Progression in Diabetic Kidney Disease (FIDELIO-DKD) trial studied the effect of finerenone, compared with placebo, in 5734 participants with type 2 diabetes and CKD. Those with an eGFR 25–60 mL/min/1.73m^2^, urine albumin-to-creatinine ratio (UACR) of 30–300 mg/g and diabetic retinopathy, or eGFR 25–75 mL/min/1.73m^2^ with UACR of 300–5000 mg/g were eligible. Compared to placebo, allocation to finerenone significantly reduced risk of the primary composite kidney disease progression outcome by 18% (hazard ratio [HR] 0.82, 95% confidence interval [CI] 0.73–0.93) [48]. The Finerenone in Reducing Cardiovascular Mortality and Morbidity in Diabetic Kidney Disease (FIGARO-DKD) trial randomized 7437 participants with type 2 diabetes, eGFR 25–60 mL/min/1.73m^2^ and UACR of 30–300 mg/g, or eGFR > 60 mL/min/1.73m^2^ with UACR of 300–5000 mg/g. Compared with placebo, allocation to finerenone significantly reduced the risk of the primary composite cardiovascular outcome by 13% (HR, 0.87 [95% CI, 0.76–0.98]) [47]. Pooled analyses of these two trials (referred to as FIDELITY) found hospitalization for HF was the cardiovascular outcome with the most clear benefit (HR, 0.78 [95% CI, 0.66–0.92]) [41]. Importantly, both these large finerenone trials excluded patients with known significant non-diabetic kidney disease. The Finerenone Trial to Investigate Efficacy and Safety Superior to Placebo in Patients with Heart Failure (FINEARTS-HF) trial compared finerenone versus placebo among 6016 participants with HF with mildly reduced or preserved ejection fraction. Finerenone reduced the risk of total worsening HF events or cardiovascular death by 16% (rate ratio, 0.84 [95% CI, 0.74–0.95]) [49]. The FINE-HEART pooled meta-analysis combining 18 991 participants from all three trials reported risk of the kidney composite outcome (defined as a ≥50% sustained decline in eGFR from baseline, decline in eGFR to <15 mL/min/1.73m^2^, kidney failure, and death from kidney failure) was reduced by 20% (HR, 0.80 [95% CI, 0.72–0.90]) [42]. These results are consistent with the known beneficial effects of steroidal MRAs (spironolactone and eplerenone) on risk of cardiovascular death or hospitalization for HF in patients with chronic HF [58–60], with steroidal MRAs demonstrated to reduce risk in people with HF with reduced ejection fraction and, non-steroidal MRAs reducing the risk in mildly reduced or preserved ejection fraction (i.e. ≥40%) [60].

Small randomized trials studying patients with CKD in the absence of diabetes have suggested MRAs reduce BP, albuminuria, and markers of kidney fibrosis [61]. The placebo-controlled FIND-CKD (FInerenone, in Addition to Standard of Care, on the Progression of Kidney Disease in Patients with Non-Diabetic Chronic Kidney Disease, NCT 05047263) trial is assessing whether finerenone 10 or 20 mg once daily slows rate of eGFR decline (based on a 32-month total eGFR slope) in 1584 patients with a non-diabetic causes of proteinuric CKD [62, 63]. Key eligibility criteria are CKD without diabetes and UACR ≥ 200, ≤3500 mg/g with eGFR ≥ 25, <90 mL/min/1.73m^2^ on a stable RASi, plus serum potassium ≤4.8 mmol/L [62].

There are limited data regarding co-administration of SGLT2i and MRAs in the large trials of CKD. CREDENCE excluded participants using an MRA [64], while in DAPA-CKD only 229 (5.3%) participants were co-prescribed MRAs [65]. In DAPA-CKD, effects on kidney disease outcomes or risk of hyperkalaemia were similar in the types of patient prescribed MRA versus those who were not [65]. Similarly, univariable subgroup analyses by baseline MRA co-prescription from several of the non-CKD trials have found that the relative risk reduction in cardiovascular events were similar in patients prescribed an MRA compared with those who were not [66–73]. The ongoing Combination effect of Finerenone and Empagliflozin in participants with CKD and type 2 diabetes using a UACR Endpoint (CONFIDENCE NCT05254002) trial is assessing the effects of a combination of finerenone and empagliflozin versus each treatment alone on UACR in 807 patients with type 2 diabetes and eGFR 30–90 mL/min/1.73m^2^ and UACR ≥ 300 and <5000 mg/g [74].

## ALDOSTERONE SYNTHASE INHIBITORS

**Figure 2:**
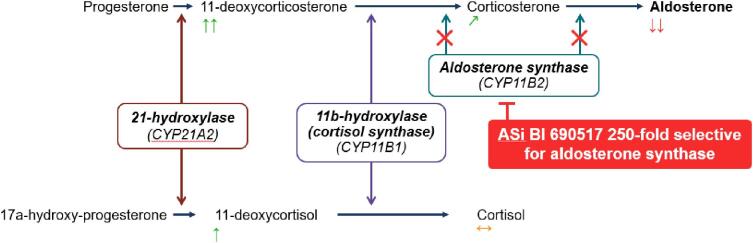
Aldosterone and cortisol synthesis pathway and the qualitative effects of inhibiting aldosterone synthase with vicadrostat (BI 690517) in patients with CKD. ASi = aldosterone synthase inhibitor. Arrows represent observed effects of vicadrostat on aldosterone and cortisol in the phase II CKD trial (1378-0005), and predicted directions of effect of vicadrostat on their circulating precursors. The comparator for the 250-fold selectivity is cortisol synthase. Hypothetically, adrenal effects of aldosterone synthase inhibition could result in no effect on serum cortisol, or could result in decreased or increased serum cortisol concentrations. Decreases may result from possible inhibition of cortisol synthase, which would inhibit conversion or 11-deoxycortisol to cortisol. Inhibition of aldosterone synthase also results in increased levels of 11-deoxycorticosterone (i.e. the shared precursor of both aldosterone and cortisol), which could lead to increased substrate for cortisol synthesis.

Aldosterone is synthesized from cholesterol [50] and the final synthesis steps are mediated by the enzyme aldosterone synthase encoded by *CYP11B2* [75]. Aldosterone synthase is the target for a new class of drugs termed aldosterone synthase inhibitors (ASi) [76]. Developing a highly selective ASi targeting *CYP11B2* is required as it shares 93% sequence homology with cortisol synthase (*CYP11B1*), a key enzyme and the final rate-limiting step in cortisol synthesis [76]. Aldosterone and cortisol share precursors, and so an ASi could theoretically cause variable effects on cortisol synthesis (i.e. increases could result if common precursors in the steroidogenesis pathway are diverted to cortisol synthesis by an ASi, or decreases could result if there is off-target inhibition of cortisol synthase [*CYP11B1*]; Fig. 2). The development of an early ASi, osilodrostat, for the treatment of hypertension was discontinued as it was also found to be a potent inhibitor of cortisol synthase resulting in decreased cortisol levels and blunted cortisol response to ACTH stimulation testing [77]. Osilodrostat has since been developed as a treatment for Cushing's syndrome [78]. Conversely, in a trial of 200 patients with uncontrolled hypertension and obesity or suppressed renin [79] the ASi lorundrostat resulted in dose-dependent reductions in systolic BP at 8 weeks, with the 50 mg once daily dose resulting in a –9.6 (95% CI, –15.8 to –3.41) mmHg difference compared with placebo [79]. Pharmacodynamic investigation revealed a modest increase in mean serum cortisol [79]. Another ASi, baxdrostat, was tested in 248 patients with treatment-resistant hypertension (defined as BP of ≥130/80 mmHg on stable doses of three other antihypertensive agents, including a diuretic) [80]. Allocation to baxdrostat resulted in dose-dependent reductions in systolic BP at 12 weeks, compared with placebo (2 mg dose –11.0 [95% CI, –16.4 to –5.5 mmHg]). No difference in mean serum cortisol levels was reported between allocated groups [80].

**Figure 3:**
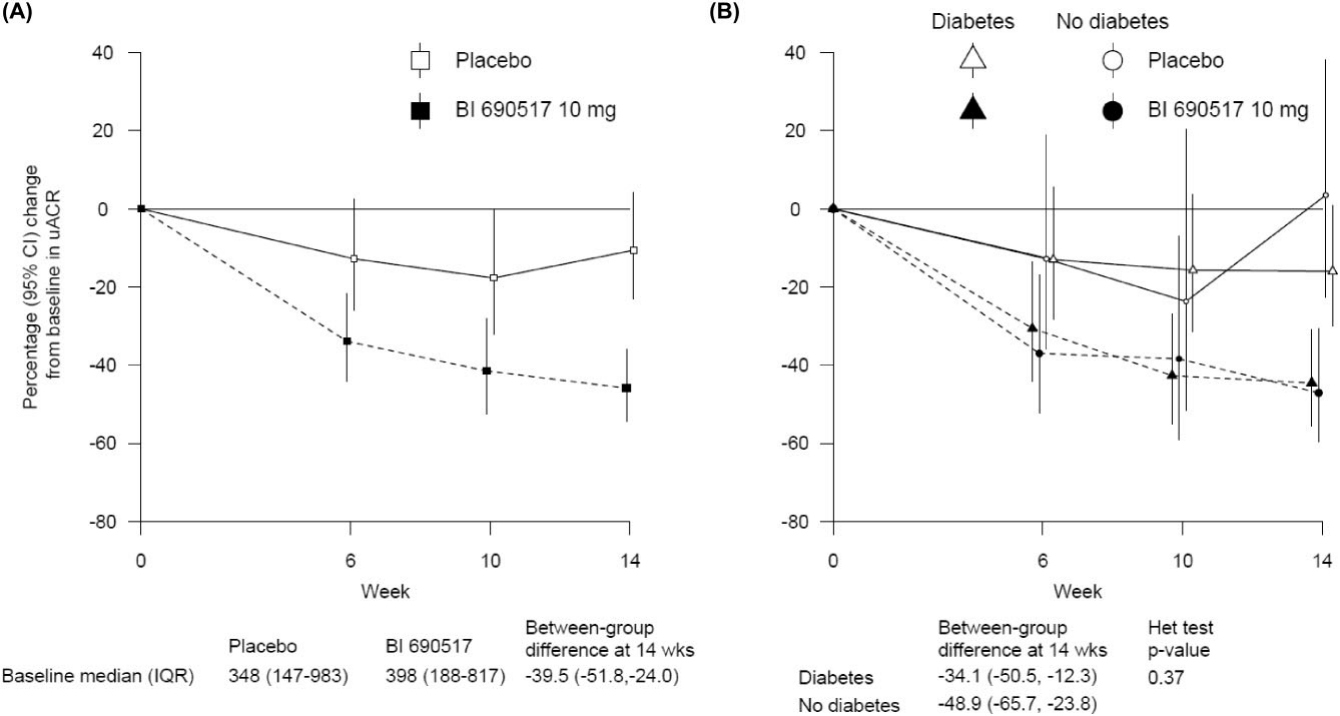
Effect of vicadrostat (BI 690517) 10 mg once daily versus placebo on urine albumin-to-creatinine ratio (UACR) when given in combination with empagliflozin overall (Fig. 3A) and by diabetes status (Fig. 3B) from the phase II CKD trial (1378-0005 post-hoc analyses). Linear mixed models for repeated measures analyses were used to estimate the difference in log transformed uACR from baseline to 14 weeks. Dotted lines are for active vicadrostat, solid lines are for placebo.

In CKD, the ASi vicadrostat (BI 690517) has been studied in a detailed dose-finding trial for CKD (1378-0005). Vicadrostat has 250-fold higher selectivity for aldosterone synthase compared with cortisol synthase [81]. The trial primarily assessed the effects of vicadrostat on change in UACR after 14 weeks in adults with eGFR ≥ 30 and <90 mL/min/1.73m^2^, and UACR ≥ 200 and <5000 mg/g, who were on stable RASi with a serum potassium ≤4.8 mmol/L. Participants were first randomized to empagliflozin versus placebo (n = 714). Subsequently, 586 participants underwent a second randomization 8 weeks later, to one of three doses of vicadrostat (3 mg, 10 mg, or 20 mg) versus matching placebo in a ratio of 1:1:1:1 (whilst continuing on the background empagliflozin or its matching placebo, as originally allocated). At baseline, mean (SD) eGFR was 51.9 (17.7) mL/min/1.73m^2^, median (interquartile range) UACR was 426 (205–889) mg/g and 71% of participants had diabetes. Among those allocated to empagliflozin, the difference in UACR versus placebo in vicadrostat dose groups was –9% for 3 mg, –40% for 10 mg, and –33% for 20 mg [81]. Vicadrostat 10 mg has been selected as the optimum dose to study in future trials of CKD. Importantly, these effects of the 10 mg dose on UACR were evident in participants with and without diabetes (Fig. 3).

The effect of vicadrostat on UACR was similar irrespective of initial allocation to empagliflozin [81], but interestingly the effect on BP was augmented by the presence of empagliflozin [81]. In combination with empagliflozin, systolic BP was 7.8 mmHg lower with vicadrostat 10 mg versus placebo [81]. Vicadrostat caused a reversible acute eGFR dip in addition to the dip resulting from initiation of empagliflozin. Vicadrostat caused a dose-dependent reduction in aldosterone exposure, with the largest difference reaching a ∼60% reduction [81]. There was no meaningful difference in mean serum cortisol levels between groups during the 14 weeks of treatment. The incidence of a morning cortisol <83 nmol/L (<3 mcg/dL) was 3.7% (16/436) in participants in the active vicadrostat groups versus 2.7% (4/147) in the placebo vicadrostat groups [81].

### Effects of aldosterone inhibition on serum potassium

**Figure 4:**
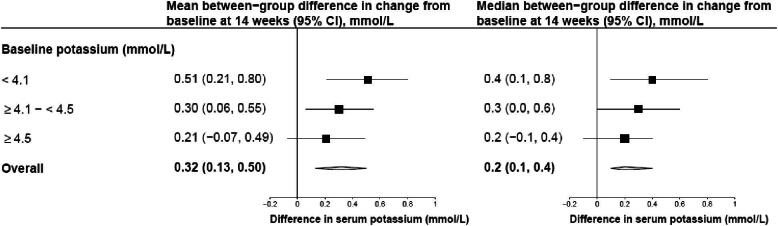
Effect of vicadrostat (BI 690517) 10 mg when combined with empagliflozin, compared with placebo on mean and median potassium concentration by baseline potassium from the phase II CKD trial (1378-0005 post-hoc analyses). *P*-value for trend across mean differences = 0.15.

Both MRAs and ASi’s increase serum potassium levels [47, 48, 79, 81]. The FIDELITY meta-analysis showed that finerenone increased potassium by 0.21 (SD 0.47) mmol/L from baseline, compared with 0.02 (SD 0.43) mmol/L with placebo [41]. Similarly, in the phase II CKD trial of vicadrostat, serum potassium was 0.32 (95% CI, 0.13, 0.50) mmol/L and 0.24 (95% CI, 0.08, 0.41) higher respectively, compared with placebo, for the 10 and 20 mg doses (when used in combination with empagliflozin) [81]. The effect of vicadrostat on potassium was similar in size when assessed separately by baseline level of potassium (i.e. the effect on potassium was no larger in those with high baseline potassium, Fig. 4).

Findings from the currently reported finerenone and ASi trials have limited their generalizability due to a serum potassium screening visit eligibility criterion requiring potassium of ≤4.8 mmol/L [41, 79, 82]. Combining a SGLT2i with inhibitors of the RAAS may help mitigate some of the risk of hyperkalaemia associated with MRAs, ACEi, or ARBs. Meta-analyses of participants with type 2 diabetes from SGLT2i trials have shown SGLT2i reduced the risk of hyperkalaemia (although there was limited data in patients treated with MRAs) [83, 84]. In the EMPA-KIDNEY trial, empagliflozin was associated with a similar sized albeit non-significant reduction in risk of serious hyperkalaemia [14]. These beneficial effects may be mediated by the modest reduction in serum potassium [14]. In the phase II CKD trial of vicadrostat, the numerically smaller increases in serum potassium with vicadrostat among those also allocated to empagliflozin were not significantly different from the group allocated placebo empagliflozin, but the trial may have been too small to detect the expected about –0.04 mmol/L predicted difference that was observed in EMPA-KIDNEY [14]. Data on follow-up RASi use from CREDENCE and DAPA-CKD, suggest SGLT2i may result in better adherence to RASi [85]. Taken together, these data begin to raise a possibility that SGLT2i may facilitate adherence to inhibitors of the RAAS (and allow for future trials to use less stringent eligibility potassium thresholds).

## THE EASi-KIDNEY TRIAL

EASi-KIDNEY (The Studies of Heart & Kidney Protection with vicadrostat) in combination with empagliflozin (NCT06531824), is a multicentre international randomized double-blind placebo-controlled clinical trial that is designed to investigate outstanding uncertainties about the efficacy and safety of interventions that target the aldosterone pathway in CKD in the era of SGLT2i use. To date, large-scale randomized data assessing MRA use in people with CKD are limited to patients with type 2 diabetes with albuminuria. Important unanswered questions include:

- What is the efficacy, safety, and tolerability of ASi when used in combination with SGLT2 inhibitors?
- What are the effects in patients underrepresented in trials of non-steroidal MRA therapy (i.e. those with a non-diabetic causes of CKD, with low levels of eGFR, with low levels of albuminuria, and/or blood potassium levels over 4.8 mmol/L)?

EASi-KIDNEY will compare the ASi vicadrostat versus matching placebo when used in combination with empagliflozin 10 mg once daily in about 11 000 patients with CKD at risk of progression. Such risk is identified using simple inclusion criteria: eGFR ≥ 20 and <45 mL/min/1.73m² regardless of albuminuria; or eGFR ≥ 45 and <90 mL/min/1.73m^2^ with UACR ≥ 200 mg/g (or protein-to-creatinine ratio ≥300 mg/g). EASi-KIDNEY will require a local investigator to confirm that each potential participant neither requires an ASi or MRA nor that such treatment is definitely inappropriate (i.e. applies the uncertainty principle), and that they agree to the use of empagliflozin. The protocol will also require that the participant is prescribed a clinically appropriate dose of a RASi, unless such treatment is either not tolerated or not indicated.

**Figure 5:**
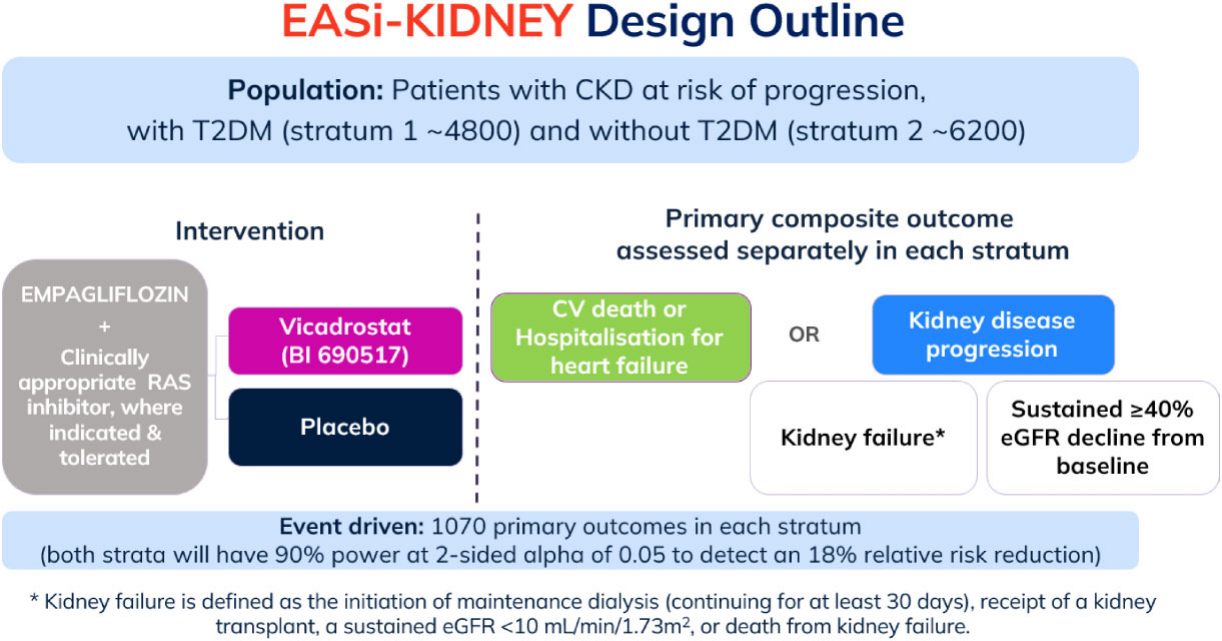
EASi-KIDNEY's double-blind placebo control design outline. Each stratum has the same design and will recruit in parallel. The sample sizes were selected in order that each stratum should separately accrue 1070 outcomes over a median follow-up of 3.0 years.

In order for EASi-KIDNEY to ensure clear results in patients with and without diabetes, its design uses two separate strata (i.e. study parts). Stratum 1 will recruit ∼4800 participants with type 2 diabetes and stratum 2 will recruit ∼6200 without diabetes, and each stratum will continue until it has reported at least 1070 primary outcomes as an event-driven trial. This approach enables each stratum to separately provide 90% power at a two-sided alpha of 0.05 to identify an 18% relative risk reduction in the composite primary outcome of kidney disease progression, hospitalization for HF, or death from cardiovascular causes (Fig. 5). This enables independent analysis of the two strata. The proportion of the different components of the outcomes will differ by stratum, with kidney disease progression outcomes being the most common in each (Supplementary Table S1).

EASi-KIDNEY's renal eligibility criteria broadly match those of EMPA-KIDNEY [14]. EASi-KIDNEY will therefore recruit a wide range of patients with CKD at risk of progression (i.e. a population approach). This strategy allowed EMPA-KIDNEY to assess efficacy and safety of empagliflozin across a wide range of eGFR, albuminuria categories and primary causes of CKD [14, 24, 86]. Some evidence of quantitative effect modification (i.e. small differences in effect in the same direction between subgroups) has been identified for the primary outcome in the EMPA-KIDNEY main analyses and in more sensitive eGFR slope-based analyses, but there has been no evidence of qualitative interactions (i.e. no evidence for differences in the direction of effect between subgroups). Benefits of SGLT2i on risk of hospitalization, intermediate markers (e.g. blood pressure and bioimpedance-measured fluid status) and on the primary outcome long-term, after study treatment discontinuation, are also consistently evident across subgroups [16, 86–90]. Once EASi-KIDNEY concludes, subgroup analyses will also address precision medicine hypotheses (e.g. whether the level of albuminuria modifies clinical effects of ASi). Reliability of subgroup analyses will be enhanced by a prespecified pooled analysis of the trial's two strata. Pooling data on over 2000 primary outcomes may provide sufficient power to assess effects on cardiovascular outcomes considered separately from kidney progression outcomes (the low number of cardiovascular outcomes in the active trial was a limitation of EMPA-KIDNEY) [14].

Safety outcomes will include assessing effects of vicadrostat on risk of hyperkalaemia, acute kidney injury, hypotension, and will include special focus on clinical and biochemical assessments of the mineralocorticoid and corticosteroid synthesis pathways throughout follow-up. There are dedicated corticosteroid substudies to understand any effects of chronic administration on cortisol and its precursors. Care of the participant will remain the responsibility of their local investigators, including management of any blood potassium and cortisol abnormalities (or supporting other local doctors with such management). Participants will carry participant cards explaining the risk of euglycaemic ketoacidosis and potential for cortisol abnormalities. Blood pressure will be measured at every visit, and site staff are trained to recognize manifestations of cortisol disorders. An early morning cortisol will be measured if there is suspicion of clinical cortisol insufficiency at any time during follow-up (in addition to scheduled early morning cortisol measurement at 1- and 3-month visits). Additional detail on the strategies for managing potassium abnormalities, cortisol disorders and blood pressure are outlined in the Supplementary materials.

To help ensure more widely generalizable results, exclusion criteria will be kept to a minimum. Modelling from the potassium levels represented in EMPA-KIDNEY and applying the observed effect of vicadrostat on potassium in the phase II CKD trial (1378-0005) suggests an exclusion criteria of a serum potassium >5.2 mmol/L may be optimum. It is predicted that such a threshold would result in an absolute excess rate of serum potassium levels of >6.0 mmol/L of 2.4% (3.1% in those allocated vicadrostat versus 0.7% among those allocated placebo; Supplementary Table S2). This is similar to the absolute excess rates of potassium >6.0 mmol/L of 3.1% from FIDELIO-DKD (placebo 1.4% versus finerenone 4.5%). The distribution of potassium in EMPA-KIDNEY participants suggests this approach should mean ∼90% of patients who were otherwise eligible can be randomized (a threshold of 4.8 mmol/L potentially excludes about a quarter of potential participants with CKD). EASi-KIDNEY local investigators will be asked to aim to maintain blood potassium below 5.5 mmol/L where possible, and to stop vicadrostat/placebo should potassium levels reach ≥6.0 mmol/L.

Methodology for large CKD trials like EASi-KIDNEY is important to refine. Widespread use of the new effective treatments for CKD should mean lower event rates in the future, resulting in trial sample sizes necessarily increasing. Small trials that study individual causes of CKD with treatments that could improve outcomes for a wider range of causes of CKD should be discouraged as a false solution to the challenge of falling event rates. Such approaches risk inadequate information on efficacy and safety on new drugs before they enter clinical practice, and premature availability of new interventions could also harm the feasibility of conducting future definitive trials in the wider range of patients who could benefit. We believe nephrology needs to continue to embrace the large simple trial designs when studying interventions that could modify final common pathways of progression or cardiovascular risk. Streamlined approaches to maximize sample size have led to major advances for cardiology and other therapeutic areas [91, 92]. CKD trials testing the efficacy and safety of baxdrostat in combination with dapagliflozin in people with CKD and high blood pressure are ongoing (e.g. NCT06268873) [93], and future collaborative meta-analysis combining data from trials of other ASi will be needed as even large trials are limited in the number of hypotheses that can be addressed reliably [93].

## SUMMARY

Patients with CKD should be offered participation in large trials of interventions that could help reduce the risks associated with CKD. Trials of SGLT2i have demonstrated that population approaches can generate important efficacy and safety information in a broad range of patients with CKD, and support the existence of modifiable final common disease pathways. Targeting residual aldosterone activity despite maximally tolerated RASi with a nonsteroidal MRA reduces risks of CKD progression and HF hospitalization in patients with CKD and type 2 diabetes (with albuminuria). Large-scale randomized trials are now needed to assess the effects of inhibiting the aldosterone pathway in less-well studied patients with CKD, including those with non-diabetic causes, those with low albuminuria, and those with the somewhat higher levels of potassium excluded from previous trials. Use of vicadrostat 10 mg once daily in patients already on an SGLT2i further reduces albuminuria by a further ∼40%, and reduces blood aldosterone exposure by ∼60%. This represents an opportunity to importantly reduce risk of kidney disease progression and target harmful cardiovascular effects of aldosterone excess. Combined use of ASi with an SGLT2i may partially mitigate some of the hyperkalaemia risk associated with aldosterone lowering, improving tolerability, and allowing a wider range of patients with CKD to be treated with inhibitors of the aldosterone pathway. These concepts provide the rationale for the EASi-KIDNEY trial, which will assess the efficacy and safety of the ASi vicadrostat in combination with empagliflozin in a broad range of patients with CKD overall and in those with and without diabetes separately.

## RIGHTS RETENTION STATEMENT: AUTHOR ACCEPTED MANUSCRIPT

For the purpose of open access, the authors have applied a Creative Commons Attribution (CC BY) licence to any Author Accepted Manuscript version arising.

## Supplementary Material

gfae263_Supplemental_File

## Data Availability

Data availability for 1378-0005 is detailed in its primary publication (reference 82).
