# Supplementary material for: The potential for improving cardio-renal outcomes in chronic kidney disease with the aldosterone synthase inhibitor vicadrostat (BI 690517): a rationale for the EASi-KIDNEY trial
Source: Nephrol Dial Transplant. 2024 Nov 12;40(6):1175–86. doi: 10.1093/ndt/gfae263 (PMC12209857; doi:10.1093/ndt/gfae263)
Supplement: gfae263_Supplemental_File [file gfae263_Supplemental_File.docx]

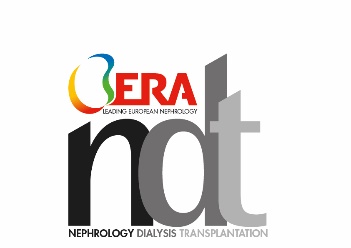


**Title: The potential for improving cardio-renal outcomes in chronic kidney disease with the aldosterone synthase inhibitor vicadrostat (BI 690517):**

**a rationale for the EASi-KIDNEY trial**

**Supplementary Materials**

Contents

[**Writing Committee** 2](#_Toc178951752)

[**Supplementary Design Detail** 3](#_Toc178951753)

[1.1 Outline summary of EASi-KIDNEY potassium, blood pressure, and cortisol management strategies 3](#_Toc178951754)

[**Supplementary Tables** 5](#_Toc178951755)

[1.2 Supplementary Table 1: Predicted number of EASi-KIDNEY participants with an outcome event by stratum once 1070 participants with a primary outcome have been reported 6](#_Toc178951756)

[1.3 Supplementary Table 2: Projected numbers of EASi-KIDNEY participants with sample above K+ threshold 7](#_Toc178951757)

# **Writing Committee**

Parminder K Judge*^1^, Katherine R Tuttle^2^, Natalie Staplin^1^, Sibylle J Hauske^3,4^, Doreen Zhu^1^, Rebecca Sardell^1^, Lisa Cronin^3^, Jennifer B Green^5^, Nikita Agrawal^1^, Ryoki Arimoto^1^, Kaitlin Mayne^1,6^, Emily Sammons^1^, Martina Brueckmann^3,7^, Shimoli V Shah^3^, Peter Rossing^8,9^ Masaomi Nangaku^10^, Martin J Landray^1^, Christoph Wanner^1,11^, Colin Baigent^1^, Richard Haynes*^1^, William G Herrington,*^1^ on behalf of the EASi-KIDNEY Steering Committee

*Equal contributions

1. Renal Studies Group, Clinical Trial Service Unit & Epidemiological Studies Unit, Nuffield Department of Population Health, University of Oxford, Oxford, UK
2. Providence Inland Northwest Health, Spokane, WA and University of Washington, Seattle, WA, US
3. Boehringer Ingelheim International GmbH, Ingelheim, Germany
4. Vth Department of Medicine, University Medical Center Mannheim, Heidelberg University, Mannheim, Germany
5. Duke Clinical Research Institute, Durham, North Carolina, US
6. School of Cardiovascular and Metabolic Health, College of Medical and Veterinary Life Sciences, University of Glasgow, Glasgow, UK
7. Ist Department of Medicine, University Medical Center Mannheim, Heidelberg University, Mannheim, Germany
8. Steno Diabetes Center Copenhagen, Copenhagen Denmark
9. Department of Clinical Medicine, University of Copenhagen, Copenhagen, Denmark
10. Division of Nephrology and Endocrinology, University of Tokyo Hospital, Tokyo, Japan
11. University Clinic of Würzburg, Germany

# **Supplementary Design Detail**

## Outline summary of easi-kidney potassium, blood pressure, and cortisol management strategies

Care of EASi-KIDNEY participants will remain the responsibility of their local investigators, including management of their blood pressure, and any blood potassium or cortisol abnormalities (with support from central clinicians)

Potassium management strategies

The aim is to maintain blood potassium levels at ≤6.0 mmol/L, and ideally ≤5.5 mmol/L. If not already done so, local Investigators will be advised to stop study treatment with vicadrostat (BI 690517) after serious hyperkalaemia or a non-haemolyzed blood potassium of 6.0 mmol/L (or mEq/L) or above. Restarting vicadrostat will be at the discretion of local investigators.

Serum potassium and bicarbonate will be measured at the sites’ local laboratory at every study visit. Participants will be established on empagliflozin at screening to ensure stable treatment by randomization. Within 3 weeks of randomization, a local check of potassium will be arranged and then again at every study visit. Study visits are scheduled at 1, 3 and 6 months after randomization and then 6 monthly until the end of follow-up. In addition, an extra visit(s) may be arranged for any participant who requires review outside of their planned visit schedule. To reduce risk of serious hyperkalaemia, local investigators could consider the following strategies to manage risk of hyperkalaemia, in accordance with local practice:

- Dietary advice (+/- local dietetic referral)
- Review of non-study drugs that might increase potassium
- Review and manage acidosis and glycaemic control
- Potassium binder treatment (depending on local availability)
- Changes to frequency of follow-up and/or blood potassium monitoring

Local investigators could organize, based on participant characteristics and blood potassium levels, increased frequency of follow-up visits and/or local laboratory monitoring of potassium.

Blood pressure management approach

Participants with symptomatic hypotension, or systolic blood pressure <100 mmHg or >180 mmHg (using local blood pressure machines), will be excluded from recruitment at screening. Blood pressure will be measured at every study visit and the management will be left to the discretion of local investigators (who are encouraged to follow relevant clinical practice guidelines). Training materials for study site staff will explain how low blood pressure can be a manifestation of clinical cortisol insufficiency.

Cortisol management approach

Early morning cortisol will be monitored at 1 and 3 months after randomization in all participants, and whenever relevant symptoms develop. Site staff will be trained on the symptoms and signs associated with cortisol disorders and when to request an additional local cortisol axis assessment. Low early morning cortisol values will trigger clinical investigation and recording of an adverse event of special interest. Those participants willing to have early morning blood testing throughout the trial will have serial assessments of cortisol and corticosteroid precursors at each follow-up (a corticosteroid surveillance substudy). Cushing’s syndrome is also an adverse event of special interest as it is unclear how chronic administration of vicadrostat might affect the cortisol axis. It is possible cortisol levels could increase, decrease or remain unchanged. A late night salivary cortisol substudy will also assess for development of corticosteroid excess.

# **Supplementary Tables**

## Supplementary Table 1: Predicted number of EASi-KIDNEY participants with an outcome event by stratum once 1070 participants with a primary outcome have been reported

| **Outcome** | **Stratum 1**  **Type 2 diabetes** | **Stratum 2**  **No Type 2 diabetes** |
| --- | --- | --- |
| **Kidney Failure**     Dialysis     Transplant     Sustained eGFR <10 | **315**  212  13  220 | **389**  248  33  301 |
| **Sustained ≥40% eGFR decline** | **709** | **901** |
| **Hospitalization for heart failure** | **276** | **108** |
| **Cardiovascular death** | **179** | **80** |
| **Participants with at least one primary outcome** | **1070** | **1070** |

*Footnote: eGFR=estimated glomerular filtration rate. The table presents the number of first primary outcome and any subsequent events, so participants with an event may contribute more than once in the table. Numbers of events predicted based on numbers of relevant outcomes from empagliflozin arm of the EMPA-KIDNEY trial scaled to 1070 (by diabetes status at baseline). The event driven design and 1070 primary outcomes will provide 90% power at 2-sided alpha=0.05 to detect an 18% relative risk reduction (i.e. a hazard ratio of 0.82) in each stratum separately. Pooled analyses of the two strata will have even better power and will assess a pre-specified composite outcome of kidney failure, hospitalization for heart failure or cardiovascular death (i.e. excluding the use of the surrogate of progression of a sustained ≥40% eGFR decline). The pooled analyses will enable the most reliable pre-specified assessment for any differences in the effects of vicadrostat (BI 690517) in patients with subcategories of primary cause of kidney disease (diabetes vs glomerular vs ischemic/hypertensive vs other/unknown), by baseline kidney function and level of albuminuria, and by diabetes status (i.e. four key subgroups).*

## Supplementary Table 2: Projected numbers of EASi-KIDNEY participants with sample above K+ threshold

| Exclusion criteria, mmol/L | Follow-up potassium threshold, mmol/L | Placebo (N=5500) | Vicadrostat (N=5500) | Absolute excess  (vicadrostat vs placebo) |
| --- | --- | --- | --- | --- |
| **>5.2** | **>5.5** | **445 (8.1%)** | **1154 (21.0%)** | **12.9%** |
|  | **>6.0** | **41 (0.7%)** | **173 (3.1%)** | **2.4%** |
| >5.1 | >5.5 | 401 (7.3%) | 1079 (19.6%) | 12.3% |
|  | >6.0 | 34 (0.6%) | 151 (2.7%) | 2.1% |
| >5.0 | >5.5 | 350 (6.4%) | 988 (18.0%) | 11.6% |
|  | >6.0 | 27 (0.5%) | 126 (2.3%) | 1.8% |
| >4.8 | >5.5 | 234 (4.2%) | 752 (13.7%) | 9.5% |
|  | >6.0 | 14 (0.2%) | 75 (1.4%) | 1.2% |

*Footnote: Potassium data at 3 visits in the first year and at 18 and 24 month visits were simulated using a multivariate normal distribution and used to estimate the expected number of participants with at least one sample that exceeds the stated potassium threshold. For the placebo arm, the assumed means and SDs of potassium at each visit and the correlations between visits were based on observed potassium values in the subset of EMPA-KIDNEY participants allocated empagliflozin whose baseline potassium value did not exceed the specified exclusion criteria. For the ASi arm, the mean potassium value at each visit was increased by 0.28 mmol/L (based on the average of the increases in potassium seen in the Phase 2 data in the two higher dose groups) but the same SDs and correlations were assumed. In FIDELIO-DKD, finerenone use resulted in a higher proportion of participants with a serum potassium of >6.0 mmol/L compared with placebo (4.5% vs 1.4%, an estimated absolute excess of 3.1%).(Bakris GL, Agarwal R, Anker SD, Pitt B, Ruilope LM, Rossing P, et al. Effect of Finerenone on Chronic Kidney Disease Outcomes in Type 2 Diabetes. N Engl J Med. 2020;383(23):2219-29.)*
